# Subacromial Impingement Syndrome: A Systematic Review of Existing Treatment Modalities to Newer Proprioceptive-Based Strategies

**DOI:** 10.7759/cureus.28405

**Published:** 2022-08-25

**Authors:** Harman Singh, Aaronvir Thind, Nequesha S Mohamed

**Affiliations:** 1 Internal Medicine, Medical University of the Americas, Nevis, KNA; 2 Orthopaedics, Wake Forest School of Medicine, Winston-Salem, USA

**Keywords:** joint position sense therapy, proprioception-based exercises, kin tape, subacromial impingement syndrome, shoulder kinematics, proprioceptive treatment

## Abstract

Musculoskeletal pain is a common reason for primary care visits, with many visits for shoulder pain due to subacromial impingement syndrome (SIS). Current treatments lack evidence for effective management, showing only temporary outcomes. This systematic review evaluates existing modalities in comparison to the use of more permanent proprioceptive-based strategies. Specifically, this meta-analysis compared the use of kinesiology tape, myofascial trigger point release (MPTR), scapular stabilization exercises (SSE), and resistance training. PubMed, BioMedCentral, and ScienceDirect databases were queried for studies evaluating proprioceptive-based exercises in the last nine years. In total, 48 studies met the inclusion and exclusion criteria. After removing duplicates, a total of 14 level 1 studies were left. Kinesiology tape use demonstrated a statistically significant reduction in pain-free range of motion. MPTR improved in all pain scores and the disability scores index. SSE also reduced pain; however, mixed results were seen for range of motion. Finally, resistance training not only reduced pain but improved proprioception and joint position sense. Even though all techniques showed some promise in treating SIS, further large-scale studies exploring related outcomes are needed.

## Introduction and background

Musculoskeletal pain is the second most common reason for visits to a primary care physician. The American Academy of Orthopaedic Surgeons (AAOS) estimates that a quarter of Americans have a musculoskeletal condition, which costs the United States over $850 billion dollars per year [1]. The prevalence of these conditions has led to a doubling of skeletal muscle relaxant prescriptions from 2005 to 2016 [2]. Of all musculoskeletal pain disorders, shoulder pain is the third most common reason for chronic pain visits [3]. The anatomy of the thoracic spine plays a crucial role in these pathologies as it is linked to different orientations of the scapula. In patients with subacromial impingement syndrome (SIS), particularly secondary SIS caused by muscular imbalance, the scapula can be found to be more protracted and the thoracic spine more flexed [4]. These alignment impairments may interfere with shoulder kinematics, leading to poor posture with chronic loss of range of motion and increased muscle relaxant prescriptions as patients attempt to deal with the pain [5].

It is estimated that 44-65% of all visits for shoulder pain are due to SIS [6]. Primary impingement syndrome is caused by structural changes that cause the narrowing of the subacromial space. Secondary impingement syndrome refers to an incorrect centering of the humeral head often due to muscular imbalance causing soft-tissue impingement when the shoulder joint is moved [3]. SIS does not describe one specific disorder but rather a spectrum of possible pathological processes, including partial thickness tears, rotator cuff tears, rotator cuff tendinosis, calcific tendinitis, and subacromial bursitis. Its prevalence is high in a wide range of repetitive overhead sports, such as swimming, volleyball, and handball, as well as in manual jobs requiring prolonged overhead positioning of the arm such as builders, electricians, and hairdressers.

The main consequences of SIS are functional loss, pain, and disability. Treatment strategies include a combination of exercise therapies, steroid injections, and, for refractory or severe patients, surgery [7]. There is growing evidence to support the use of resistance training, improved joint position sense, and proprioceptive shoulder exercises over movement-based exercise therapies alone. Current research, however, not only lacks evidence for the outcome of these management modalities but, specifically, there is a limitation as to which exercise therapies are most clinically effective [8].

There remains a need for high-quality clinical research on the treatment of SIS. This systematic review will focus on evaluating several existing functional rehabilitation strategies in comparison to the use of specific proprioceptive-based strategies. It also reviews scapular kinematic deficits that should also be addressed with specific exercises in the rehabilitation of SIS.

## Review

Methodology

Databases Queried

Following Preferred Reporting Items for Systematic Reviews and Meta-Analyses (PRISMA) guidelines, a systematic review of the literature for proprioceptive-based exercise therapies was conducted by searching PubMed, BioMedCentral, and ScienceDirect. Articles published in the past nine years (January 1, 2011, to December 31, 2020) were identified using various keyword combinations. The following string was utilized for the search: ((“Subacromial impingement syndrome” OR “SIS” OR “Chronic Shoulder pain”) AND (“Kinesiology tape” OR “KT” OR “Scapular stabilization exercises” OR “SSE” OR “Resistance training” OR “Myofascial Trigger point release” OR “MTPR”)).

Inclusion and Exclusion Criteria

Publications were eligible for inclusion if they: (1) included patients with existing chronic shoulder pain and/or SIS; (2) included a comparison of both pre and posttreatment results; (3) the level of evidence was level 2 or higher based on the American Society of Plastic Surgeons; and (4) the study was performed in the United States, Canada, United Kingdom, or Australia. Studies were excluded if they: (1) were published beyond 2011; (2) included surgical intervention as a treatment modality; (3) were written or published in a language other than English; (4) the full text was not available; and (5) were systematic reviews, meta-analyses, case reports, case studies, feasibility or pilot studies, letters to the editor, or surveys.

Eligible Studies

**Figure 1:**
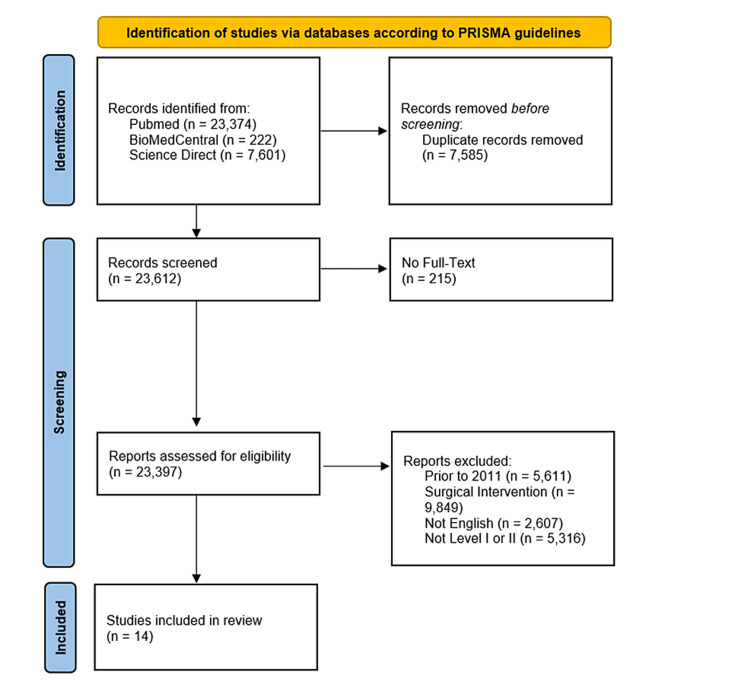
PRISMA flowchart. PRISMA: Preferred Reporting Items for Systematic Reviews and Meta-Analyses

The primary search of the PubMed database generated 23,374 results, of which 25 met the inclusion and exclusion criteria. The original search of the BioMed database resulted in 222 entries. Of these, seven met the inclusion and exclusion criteria. The initial search of the ScienceDirect database returned 7,601 results, of which 16 met our inclusion and exclusion criteria. After removing duplicates and further assessing for relevance, we were left with a total of 14 studies, all reporting level 1 evidence (Figure 1).

Study Data/Extracted Data

The 14 studies included in this systematic review were assessed, and the data extracted included the type of study, diagnosis, type of treatments utilized, exercise therapy strategies, rehabilitation intervention, control and intervention group characteristics, time to follow-up, and outcome measures.

Treatment Modalities

The first treatment considered was kinesiology tape (KT). KT works by extending from a muscle’s origin to insertion using different degrees of stretch at either side of the tape to achieve a desired effect depending on the specific muscle ailment that it is being used for.

The second treatment, myofascial trigger point release (MTPR), involves the use of the therapist’s hands to palpate and identify points of resistance in the muscle tissue. Once found, the therapist applies specific pressure to the point until there is a release of tension or decline in pain.

Scapular stabilization exercises (SSE) refer to several exercises designed to strengthen the muscles that anchor the scapula to the thoracic cage. The serratus anterior, serratus posterior, trapezius, rhomboids, teres major, levator scapulae, and the latissimus dorsi are all crucial to allow for scapular stability which directly impacts the ability of the shoulder joint to correctly function.

Resistance training consists of specific movements and exercises that target progressive stretching/strengthening designed to reverse specific shoulder complications. This treatment modality utilizes resistance/weight training and focuses on the strengthening of muscles surrounding the shoulder joint with the intention of facilitating greater joint stabilization.

Results

Treatment One: Elastic Kinesiology Tape

When a muscle is acutely overstressed, the goal of therapy is to inhibit the muscle to decrease the load, and thereby the pain that patients feel. Elastic KT can achieve this effect by tensing the KT to 15-25% stretch starting from the painful area at the muscle insertion and ending at the origin of the muscle. In a weakened muscle, the aim is to stimulate the muscle by applying 15-25% tension to the KT and attaching it to the origin of the muscle, with the other end of the KT attached to the insertion. With these two methods, KT decreases pain and increases range of motion (ROM) [9]. Elastic KT is a useful technique that is designed to prevent and treat many musculoskeletal injuries, as well as increase sports performance [9]. Three level 1 studies evaluating the use of KT are included in this review.

Shakeri et al. [10] evaluated 30 patients with SIS. The experimental group received KT taping on/around their shoulder girdle on day one and were assessed using outcome measures, such as the Visual Analog Scale (VAS) and pain-free ROM for abduction, flexion, and scapular plane elevation. Patients kept the KT on for three days and underwent the first evaluation on day four after the KT was removed. The application of the tape was repeated, kept on for three days, and following KT removal, patients were again evaluated. A control group received taping at the same intervals but placebo taping techniques were used in place of KT. When compared with pretreatment scores, the experimental group saw a significant decrease of 2-3 points in VAS for pain intensity during movement (p = 0.000). There was a significant decrease of 3-4 points in VAS for nocturnal pain (p = 0.000). Significant differences in pain-free ROM compared to pretreatment were reported for all three shoulder ROMs (10-19 degrees) (p = 0.000). After placebo taping, the control group showed no significant differences in VAS for pain intensity during movement nor in shoulder flexion ROM when compared to pretreatment scores. A significant difference in VAS for nocturnal pain was found immediately after taping (1-point difference) and a week after taping (2-point difference). A significant difference was found in pain-free shoulder abduction ROM (9 degrees) and scapular elevation ROM (8 degrees) after one week of placebo when compared to pretreatment values (control group).

The study by Kul and Ugur [11] divided 40 patients with SIS into two groups based on the treatment modality they were to receive. The first group, KT group (KTG), received KT as well as a home exercise program (HEP). The second group, physiotherapy (PT) modalities group (PTG), received 15 sessions of physical therapy with HEP. Patients were followed up with two calls at five-day intervals for a total of six calls. Patients who had received corticosteroid injections in the last three months were not included in this study. Outcome measurements in this study included the VAS for rest, nocturnal and activity pain, as well as ROM values for active flexion, abduction, and internal rotation. Patients were assessed pretreatment (time 1; T1), after treatment (T2), and one month after treatment ended (T3). All values at T2 in the KTG showed significant changes when compared to baseline (all p < 0.001). At T3, significant improvements were seen in VAS rest pain (p < 0.01), VAS nocturnal pain (p < 0.01), and VAS activity pain (p < 0.05). The PTG showed significant improvements for all variables at T2 (p < 0.01). At T3, significant improvements were seen in shoulder abduction ROM (p < 0.05) and VAS nocturnal pain (p < 0.05). PT was more effective than KT in VAS activity pain (p < 0.05) and VAS nocturnal pain (p < 0.01) at T2 compared to T1. PTG improvements continued to be statistically significantly different from KTG until T3 for rest pain (p < 0.05).

A study by Goksu et al. [12] compared the therapeutic effects of KT versus subacromial corticosteroid injections (SCI) in patients with SIS. In total, 61 patients were separated into two groups. The KTG received taping three times in three-day intervals. The corticosteroid injection group (CIG) received a corticosteroid as well as a local anesthetic (bupivacaine). Both groups were prescribed the same home exercise regimen to follow for seven sessions with 24 hours between each session. The outcome measurements for this study evaluated flexion/abduction ROM values, and the VAS was used to quantify shoulder pain at rest/during movement. Shoulder functional status was detected by the Shoulder Pain and Disability Index (SPADI). Evaluations were done at baseline (T1), one week after therapy (T2), and four weeks after therapy (T3). Both groups were found to have significant improvements in ROM, VAS scores, and SPADI scores at the end of T2 and T3. When comparing the two groups at T2, the CIG had statistically significant improvement in VAS scores at rest (p < 0.025), abduction ROM (p < 0.028), and SPADI scores (p < 0.043). At T3, the CIG again had statistically significant changes when compared to the KTG for VAS scores at rest (p < 0.01), abduction ROM (p < 0.043), and SPADI scores (p < 0.031). Both groups had similar score improvements in VAS pain scores in motion, as well as ROM for flexion and abduction. All parameters improved after both treatment modalities at a statistically significant level.

Summary: Elastic KT has been shown to be effective in the treatment of shoulder pain, and more specifically in SIS. The VAS was an outcome measure common to all three studies assessed. KT was found to decrease VAS scores at rest, during movement, and at night. This effect lasted for at least one month after treatment ceased. Shakeri et al. found that KT increased the pain-free ROM for abduction, flexion, and scapular plane elevation. Kul and Uger found significant increases in ROM for all movements; however, this was only found immediately after treatment and not at one-month post treatment. Goksu et al., however, showed statistically significant lasting effects (four weeks post treatment) of KT on flexion and abduction ROM as well as on SPADI scores. Elastic KT appears to be an effective treatment modality for chronic shoulder pain due to SIS.

Treatment Two: Myofascial Trigger Point Release

Myofascial trigger points (MTPs) are tender points in tight bands of muscle that cause pain, known as myofascial pain. In MTPR, a therapist applies pressure on a patient’s muscle until they find an area of increase in tissue resistance, the MTP. On palpation of the MTP, the patient often experiences pain/discomfort. The pressure is maintained until the patient feels a release of tension/decline in pain or until the therapist feels a release of tension underneath their palpating finger. With this manual therapy, the practitioner searches for MTPs and attempts to provide relief at these points. Three studies reporting level 1 evidence were included to evaluate MTPR.

Bron et al. [13] investigated the effect of MTPR in patients with chronic shoulder pain. In total, 72 patients were included in the study and placed into one of two groups. The intervention group (IG) consisted of 37 patients who received MTPR treatment once weekly for a maximum of 12 weeks. Patients in the control group were instructed to continue their current treatment interventions, whether medicine or stretching. At six and 12 weeks of treatment, both groups reported results. The primary outcome measurement was the Disabilities of Arm, Shoulder and Hand (DASH) questionnaire score. Another outcome measure was the VAS for Pain (VAS-P). This is a general pain score and rates pain at that moment (VAS-P1), pain in the last seven days (VAS-P2), and the most severe pain in the last seven days (VAS-P3). Lastly, the number of muscles with MTP between the two groups was assessed and compared at six and 12 weeks. There was a significant improvement in the IG when compared to the control group at 12 weeks (all p < 0.05). Differences were detected on the DASH (mean difference = 7.7), the VAS-P1 (mean difference = 13.8), the VAS-P2 (mean difference = 10.2), and the VAS-P3 (mean difference = 13.8). The IG had a mean difference of 2.7 fewer muscles with MTPs when compared to the control group. After 12 weeks of study, 55% of IG patients reported improvement in shoulder pain, whereas this number was only 14% in the control group.

Myofascial pain syndrome (MPS) is a musculoskeletal disorder that features many MTPs, as well as increasing muscle stiffness. A study by Kisilewicz et al. [14] studied the effects of MTPR on trapezius muscle stiffness and the resultant presence of MPS. The study considered 12 professional Polish basketball players with unilateral neck or shoulder pain on the dominant side. Once MTPs were localized, they were treated with MTPR. The main outcome measure of this study was dynamic stiffness. Dynamic stiffness is the resistance of soft tissue to an external or internal force. The more dynamic stiffness, the more resistance, resulting in greater pain. Dynamic stiffness was measured immediately before and after MTPR with a device called the MyotonPRO. The results of the trial produced mixed results. There was a significant decrease in muscle stiffness of the upper trapezius by 11.8% (p < 0.01). Comparatively, no significant changes were detected in the middle or lower trapezius. Furthermore, no significant change was seen in the dynamic stiffness in the whole contralateral trapezius muscle (p > 0.05).

A study by Gordon et al. [15] examined the effects of MTPR on 23 patients with shoulder pain. All patients received four 10-minute sessions of therapy exclusively on the painful shoulder over a span of two weeks. Outcomes were assessed before treatment (T1), after two weeks (T2), and after six weeks (T3). The MyotonPRO was utilized in this study to assess changes in muscle stiffness. Muscle stiffness scores showed significant improvements for the treatment when comparing pre and posttreatment values (p = 0.012). The non-treated side did not show these same significant improvements in muscle stiffness (p = 0.241). Pain scores were assessed using the Brief Pain Index (BPI). The BPI scores showed that MTPR brought forth statistically significant changes in BPI scores (p < 0.0001). Pain scores also remained stable at the four-week follow-up appointment and continued to be stable at the 13-month follow-up appointment. The Wilcoxon test was utilized to determine three different parameters indicating the effect of MTPR on quality of life. Levels of stress (p = 0.024), average suffering (p < 0.0001), and reduction of quality of life scores (p < 0.0001) significantly improved, indicating that MTPR was effective in decreasing patient stress, decreasing patient suffering, and improving the quality of life.

Summary: MTPR was found to be an effective therapy for reducing pain, decreasing muscle stiffness, as well as improving patient quality of life and disability. Bron et al. reported that the IG that received treatment improved significantly in all pain scores when compared to the control group. Furthermore, disability scores were also seen to improve. Kisilwecz et al. identified that MTPR was not as effective in the treatment of the middle or lower trapezius regarding changes in muscle stiffness. However, the upper trapezius was responsive to MTPR, and dynamic muscle stiffness was found to decrease in post versus pretreatment scores. Gordon et al. found that all outcomes assessed showed statistically significant improvement in comparison to pretreatment scores. As exemplified above, MTPR appears to be an effective treatment for chronic shoulder pain from SIS or by other causes.

Treatment Three: Scapular Stabilization Exercises

Forward head posture and round shoulder posture are two of the most common postural disorders, often seen together. It increases the gravitational force exerted on the head, which can lead to degenerative changes in the cervical spine. This is known to be from a dysfunction of the flexion-relaxation phenomenon (FRP). The FRP is a normal and physiologic pattern that refers to the reduction or silence of myoelectric activity of the lumbar erector spinae (ES) muscle during full-trunk flexion [16]. In patients with shoulder injuries, postural correction can be seen to improve pain. Several investigations have shown that pain can be reduced through SSE. These SSEs include chin-tuck, overhead press, horizontal pull apart, chest press, serratus anterior punches, retraction plus external rotation, and scapular protraction. Some studies reported increased ROM using these exercises, which occurs from improved joint position sense and proprioception [16]. Four level 1 studies evaluating the use of SSE were included in this systematic review.

Shiravi et al. [17] assessed 132 consecutive patients who presented with secondary SIS due to forward head and round shoulder postures. All participants were submitted to the evaluation of the joint position sense (JPS) at 30, 60, 90, 120, and 150 degrees of shoulder forward flexion during the sitting position. Study group 1 consisted of 45 patients who used SSE and the control group included 45 patients who underwent no intervention. The SSEs were performed 30 minutes each day for six weeks (three sessions per week). The study found that group one had significant decreases in pain (−3.8 ± 0.48, p = 0.021) and proprioception (−2.5 ± 0.2, p = 0.033) after six weeks. The addition of SSE for the cervical spine led to greater improvements in pain, posture, FRP, and strength (start of the concentric contraction, p = 0.009, and end of the concentric contraction, p = 0.044). No significant changes were seen in pain and proprioception in the control group.

Hotta et al. [18] assessed 50 patients with SIS, of whom 25 were in the control group and 25 were in the treatment group. The treatment group underwent eight weeks of SSE with periscapular strengthening. Scapular kinematics, shoulder pain, and shoulder disability index were the outcome measures used. The orientation and position of the thorax, scapula, and humerus of the patients were assessed using the three-dimensional motion capture system 3 SPACE Liberty. Electromagnetic sensors were used, which were attached to the body segments to be analyzed and to digitize the anatomical points. There was a significant improvement in shoulder pain and disability index (p < 0.01), shoulder kinematics for upward rotation (p < 0.01), anterior tilt (p < 0.01), and internal rotation (p < 0.01) of the scapula. Muscular strength increased in the treated group after carrying out the protocol. In the treatment group, a significant reduction in pain was seen with a mean difference of 32.4 points (p < 0.01), indicating improved shoulder function in the treatment group.

Moezy et al. [19] conducted a randomized controlled trial (RCT) to compare the effectiveness of SSE with conventional PT in 68 patients with SIS. The flexibility exercises included the sleeper stretch, crossed arm stretch, and corner stretch. The outcomes measured included improved ROM and joint position sense. Scapular clock exercises using a ball were used to help with joint kinesthesia. The PT protocol included pendulum and ROM exercises. The improvement of shoulder abduction (p = 0.024), external rotation ranges (p = 0.001), postural parameters such as forward shoulder translation (p < 0.0001), forward head posture (p = 0.001), mid-thoracic curve (p = 0.001), and pectoralis minor length in the SSE group were significantly greater than that the PT group. After six weeks, the SSE group also demonstrated significant improvement in shoulder flexibility (p < 0.0001) and protraction of the shoulder (p = 0.001). In the PT group, there were also significant differences in scapular rotation and pectoralis minor length; however, no improvement in scapular symmetry and no reduction in pain were seen (p = 0.576).

Struyf et al. [20] conducted an RCT among 22 patients with SIS. The scapular-focused treatment group included stretching and scapular motor control training which included upward and downward rotation, external and internal rotation, and posterior and anterior tilting of the scapula. The control therapy group included stretching and rotator cuff training with an elastic band. The forward posture head was measured vertically with a sliding caliper. One gravity-referenced inclinometer was used to measure humeral elevation, and a second inclinometer was used to reliably measure the upward rotation of the scapula. Clinically significant improvement was seen in scapular motor control training using self-reported disability (Cohen’s p = 0.93, p = 0.025), and improvement in pain during the Neer test, Hawkins test, and empty can test (p = 0.076, 0.014, and 0.092, respectively). The experimental group demonstrated a moderate improvement in self-experienced pain at rest, whereas the control group showed no improvement. However, no significant difference was seen in the scapular upward rotation and the shoulder disability questionnaire.

Summary: The use of SSE in patients with SIS has demonstrated improvements in various outcomes of measures. With the studies evaluated for this treatment, some contradicting results were found. A key finding that was common to all the RCTs studied here was an improvement in scapular rotation and ROM. This increase in ROM can be attributed to reduced pain which was also seen in all studies. The study by Struyf et al. was unique in that no significant difference was found in scapular upward rotation; however, motor control training including external/internal rotation and posterior/anterior tilting of the scapula demonstrated improvement. In addition, all the above studies showed a reduction in pain using scapular exercises alone except the study by Moezy et al., which showed an equal reduction in pain using both SSE and PT.

Treatment Four: Resistance Training Exercises

Several studies measure the effect of active exercises and strength training for shoulder injuries and pain that cause the weakening of the surrounding muscles. Shoulder movement is a modifiable factor that can contribute to shoulder pain and disability. Because people with SIS, rotator cuff injuries, or even diabetes demonstrate decreased shoulder motion and strength, specific movement and exercise strategies targeting progressive stretching and strengthening will help to reverse these shoulder complications. Two main aspects should be taken into account during strength training: specific muscle-force level and the force balance among muscles that act on the same joint [7]. Proprioception, the ability to recognize and locate the body in relation to its position and orientation in space, is essential for motor control and joint stability during daily activities and sports practice [7]. Several studies have described its effects on muscle strengthening which directly affects the functional capacity. Therefore, it is important to understand the effects of resistance training on proprioception so that we can improve the strength-training protocols to increase joint stability. The strength-training program exercises reviewed in these studies included a sling suspension system, bench press, lat pull-down, shoulder press, seated row, inferior glide, isometric low row, dynamic knee push-up, wall press, and wall slide with weights. Four level 1 studies were included in this exercise.

Jung et al. [21] assessed 36 patients who received active shoulder exercise with a sling suspension system and 18 patients in the control group who received bilateral arm training for 40 minutes five days a week for four weeks. The outcome measures before and after the intervention included measurement of shoulder subluxation distance, shoulder proprioception, the Fugl-Meyer assessment (FMA) scale, and the manual function test (MFT). A sling suspension-based exercise method can compensate for gravity by hanging part of the body on a string. It can induce selective active muscle contraction by adjusting the gravity, designed to strengthen muscles around the shoulder joint. The control group underwent shoulder flexion-extension exercise, elbow joint flexion-extension exercise, and a forward-reaching exercise. The shoulder subluxation distance was evaluated using an L-shaped thermoplastic rod (or jig). The assessment of shoulder proprioception was performed using a repositioning test of shoulder flexion position sense using five specified angles. The FMA tool was used for quantitative assessment of the functional recovery. The change in distance measured in shoulder subluxation (p = 0.008), the degree of shoulder proprioception (p = 0.006), and the upper extremity manual function (p = 0.002) demonstrated significantly greater results in the study group than in the control group.

Shiravi et al. [17] assessed 132 consecutive patients who presented with secondary SIS due to forward head and round shoulder postures. Study group one consisted of 45 patients who used abdominal control feedback (ACF) exercises and the control group included 45 patients who underwent no intervention. All participants were submitted to the evaluation of the JPS at 30, 60, 90, 120, and 150 degrees of shoulder forward flexion during the sitting position. Shoulder proprioception was measured by a goniometer. Electromyography data were normalized for maximum voluntary contraction. The maximal isometric strength of scapular upward rotators was measured using a handheld dynamometer. The addition of ACF to a conservative program for a shoulder injury led to greater improvements in neck pain, posture, FRP, and strength. The study found that group one had significant decreases in pain (p = 0.036) and proprioception error (p = 0.034) after six weeks. No significant changes were seen in pain and proprioception in the control group.

Salles et al. [22] assessed a total of 90 male undergraduates. They were randomly distributed into three groups: group one with 24 subjects performed four exercises at the same high intensity, group two with 27 subjects performed exercises at different intensities, and the control group with 30 subjects performed no upper body exercise. The ACF exercises including bench press, lat pulldown, shoulder press, and seated row were performed 30 minutes each day for six weeks (three sessions per week). They determined the ROM for shoulder rotation by measuring the amplitude between the maximum internal and external rotation. The JPS absolute error (AE) was assessed by applying the joint-position reproduction test, with a target position at 50% of ROM. At pretraining, there was no difference in JPS AE among groups, yet at post-training, group one demonstrated less AE than both group two and the control group with the best performance. JPS improved in group one compared to group two and the AE in group two was also less compared with the control group. Meanwhile, the control group maintained the same AE and did not improve proprioceptive acuity. The results demonstrate that AE depends on training intensity; strength training improved healthy participants’ ability to reproduce joint position and thus improved proprioception.

Mueller et al. [23], conducted an RCT for three months on 52 participants with shoulder pain or limited motion and were randomized to a group receiving progressive shoulder movement intervention (ShoMo group) and a control group receiving wellness activities. The ShoMo intervention group included exercises to improve shoulder ROM. Participants started with passive stretching of end-range shoulder flexion and rotation (internal, external) that progressed to active, followed by resisted shoulder motions tailored to their ability level. Participants were then instructed to perform three assigned stretching motions for a minimum of two sets of 10 repetitions every day. Participants were also instructed in active shoulder movement that could be incorporated into daily activities with a dose based on the participant’s measured activity count at baseline using accelerometers. The intent of the wellness program was to control interactions with physical therapists (participants seen four times over three months) and to provide useful information for disease management, but not provide intervention that directly targeted shoulder joint motion. The outcome measures involved ROM and SPADI. The ShoMo group had a 7.2-degree increase in active shoulder flexion compared to the wellness group after three months of intervention (p < 0.05). However, the difference did not persist for more than three months. The ShoMo group showed a 12.7-point improvement in the total SPADI score compared to the wellness group following three months of intervention. The significant difference between groups persisted over 12 months.

Summary: The use of resistance for strengthening and active weight-bearing exercises has been shown to significantly reduce pain and improve proprioception in those who have shoulder injuries. In each of the studies reviewed, strengthening exercises improved joint position sense and shoulder kinesthesia. The simultaneous reduction in pain parallels the improved ROM also seen in the literature. In the study by Mueller et al., the results did not persist over the long term because there was no follow-up after the therapeutic intervention was completed. It becomes important to continue to note any confounding factors that may have caused the results. Therefore, the results of the present study should be verified by additional studies with larger sample sizes. Furthermore, although all the studies demonstrated improved ROM, only the study by Jung et al. actually measured the improved manual function test.

Discussion

Chronic shoulder pain is an extremely prevalent problem that plagues Americans. It is the third most common reason for physician visits, of which secondary muscular SIS pathologies make up approximately half. To assess the usefulness of specific proprioceptive-based treatment modalities compared to more traditional and existing rehabilitation exercises, this review looked at the use of KT, MTPR, SSE, as well as resistance training. KT was seen to effectively reduce pain and improve functional limitation, especially in combination with exercise therapy [11]. However, it was also seen that proper posture and scapular stabilizer exercises appear to be more effective than general exercise therapy. Furthermore, when conventional treatment modalities fail, surgical methods are considered. Yet, several RCTs have reported no difference in pain outcomes between conservative compared to surgically treated patients [24]. Aside from these options, however, this review reviewed more unconventional therapies. KT appears to be an effective treatment modality for chronic shoulder pain and improvement in functional ROM for up to four weeks posttreatment. MTPR was universally found to be an effective therapy for reducing pain, decreasing muscle stiffness, as well as improving patient quality of life. SSE-based treatment has some contradicting results where pain and ROM were improved in all studies except one. Finally, resistance training improved joint position sense and shoulder kinesthesia in each of the studies reviewed; however, the pain was less studied. All techniques reviewed showed promise in effectively treating SIS, but further studies are needed to make definitive conclusions.

There are some limitations to this review. One limitation of this review includes inconsistent patient follow-up. Most literature studies measured outcomes up to three months to a maximum of one year. To determine promising long-term results, it is vital to maintain continuity. However, the fact that not many papers have focused on this aspect of the research is the reason why this review is important. Despite the positive results seen in this paper, physicians limit SIS treatment to the more commonly used methods such as non-steroidal anti-inflammatory drugs (NSAIDs) and corticosteroids because there is a need for continuity. Another limitation is the limited number of studies available for each technique. There is limited research conducted on proprioceptive-based modalities specifically, which is the basis of this paper. Improving joint position sense is what allows for an increase in ROM. Limited ROM is the root cause of pain and functional disability. More studies are needed to help assess whether treatment modalities such as KT and resistance training increase the degree of joint position sense. The ideal goal is that once more research is conducted and shows promising results, management of SIS can shift primarily to these treatments instead of NSAIDs or just physiotherapy. Finally, the scoring systems for each outcome measured can be hard to quantify because a wide range of scoring techniques have been used. For example, studies used a varying range of exercise sets and repetitions for the resistance training group. It is important to assess the numerical threshold after which improvement was seen. This can help us determine whether continuing treatment in those that did not see improvement might have proved beneficial. Regardless of this, the reports reviewed showed promising results and allow us to potentially standardize scoring systems in the future. 

Previous literature studies have shown a range of improvements with KT treatment. Yam et al. [25] compared RCTs measuring lower limb muscle strength and performance in patients with muscle fatigue, chronic musculoskeletal disease, those without disabilities, and those with postoperative orthopedic conditions. This was done by conducting distance in a single leg hop and vertical jump height. From each study, the greatest improvement seen favoring KT was in those with chronic musculoskeletal diseases [25]. Similarly, Wilson et al. [26] saw improvements in stability for lateral ankle sprains with a reduction in recurrence seen using KT. They measured proprioception using the dynamic balance test and endurance using the heel raise test. It was seen that KT may increase afferent input and improve proprioception on ankle stability. It was also seen that KT increased plantar flexor endurance and vertical jump height. Finally, improvements in postural control were also found [26]. These results are similar to those reviewed in this study.

Literature supports MTPR treatment of head and neck muscles in tension-type headaches and migraine-type headaches. Dry needling is a type of treatment using a thin filiform needle to penetrate the skin that stimulates MTPR. In a review by Navarro-Santana et al. [27], it was seen to improve pain-related disability compared to no treatment. Similarly, Falsiroli Maistrello et al. [28] have shown the effectiveness of manual MTPR regarding frequency, intensity, and duration of attacks in both tension-type and migraine headaches. Those with either of these have a greater number of trigger points compared to healthy subjects, and a higher number correlates with the severity and the duration of attacks. The treatment used ischemic compression, myofascial release, muscle energy, soft-tissue treatment, and positional release. The results showed a greater reduction in pain, intensity, and duration scales of headaches [28].

Studies have shown that targeted SSE can lead to improvement in posture and pain. An RCT by Kang et al. [29] used 14 exercises, including press-ups in a chain, push-up plus, supine deep breathing, supine shoulder at 90 degrees of flexion with scapular protraction/retraction, arm raise in the quadruped position, lateral arm raises with 2 kg dumbbells, posture education, prone I, prone Y, prone T, prone W, and lateral pulldown. Significant improvements were seen using the pain scale and neck function using the neck disability index. An RCT was also conducted by Beurskens et al. [30] to measure the effectiveness of physiotherapy following breast cancer and axillary node dissection. Treatments included postural correction, upper extremity coordination exercise, strengthening and conditioning exercise, and exercise for lymphedema. The program took place in nine sessions over three months. The main outcome was the VAS, which showed that shoulder/arm pain was significantly improved [30].

Previous studies have shown that intensity resistance training exercises improve pain and functional mobility. The scientific evidence behind this is due to muscle hypertrophy which contributes to muscle growth that stems from an increase in neural adaptations from exercising [31]. An RCT by Jones et al. [32] was conducted to compare resistance training versus general exercises versus no treatment in women with fibromyalgia. The regime consisted of resistance training using hand weights up to 3 lb and elastic tubing. The outcomes showed improvements of 26% in multidimensional function, 15.9% in self-reported physical function, 44.6% in pain, 12.6% in tenderness, and 25% in muscle strength in the resistance groups. Similarly, a meta-analysis of 667 articles by Papa et al. [33] showed that resistance training can decrease age-related regression in functional mobility. The training focused on the large muscle groups in the lower extremities, the effects of full-body resistance training, as well as resistance training for the muscles in the core of the body, including abdominals and spine stabilizers. Improvements in the functional mobility, gait, speed, and balance of older adults were seen. The most common outcomes included the Timed Up and Go test (TUG) and the Functional Reach test (FR). There is a 15% decrease in muscle strength every 10 years after the age of 50. However, this paper shows that resistance training can slow the loss of muscle mass and muscle strength if performed two to three days per week. These findings are in line with the ones discussed here and may provide promising results in the future.

## Conclusions

Musculoskeletal disorders, and specifically SIS, are common reasons for primary care visits, and more permanent therapeutic modalities in this area need a focus of care. Most current treatment options are temporary or only have short-term outcomes. The need for improvements in care has shed light on newer therapies for treating SIS. This meta-analysis compared the use of KT, MTPR, SSE, and resistance training. All techniques reviewed showed some promise in effectively treating SIS, but further information is needed to make definitive conclusions. Future studies should explore the use of resistance, improved joint position sense, and proprioceptive shoulder exercises, and focus on providing further information and insights on the fascial mobilization used in these techniques that contribute to the outcome measures.
